# Evidence for a downward secular trend in age of menarche in a rural Gambian population

**DOI:** 10.3109/03014461003727606

**Published:** 2010-05-14

**Authors:** Sarah Prentice, Antony J. Fulford, Landing M. A. Jarjou, Gail R. Goldberg, Ann Prentice

**Affiliations:** ^a^ MRC Human Nutrition Research, Elsie Widdowson Laboratory, Cambridge, UK; ^b^ MRC Keneba, PO Box 273, The Gambia; ^c^ MRC International Nutrition Group, Nutrition & Public Health Intervention Research Unit, London School of Hygiene & Tropical Medicine, London, UK

**Keywords:** Menarche, age, Gambia, secular trend, interval regression

## Abstract

Menarcheal age is a key indicator of female maturity and development. Studies in many countries have reported a downward secular trend in age of menarche over the past century. This study presents data gained using the ‘status quo’ method and interval regression to estimate median menarcheal age of girls in a rural Gambian community. Cross-sectional studies carried out in 1989, 2000 and 2008 revealed a median menarcheal age of 16.06 (95% CI 15.67–16.45), 15.03 (95% CI 14.76–15.30) and 14.90 (95% CI 14.52–15.28), respectively. The average rate of decline of median age of menarche was amongst the most rapid yet reported, at 0.65 years of age per decade (*p* < 0.00001). There was no evidence for a change in the rate of decline over the two decades studied. These results probably reflect ongoing socio-economic development within the region.

## Introduction

Onset of menstruation is an important indicator of female maturation. It has been shown to be influenced by a range of genetic (Loesch et al. 1995), environmental and socio-economic factors including: Early life events (Liestol 1982), current body weight and height (Frisch and Revelle 1971), health (Thomas et al. 2001), nutritional intake (Simondon et al. 1997), physical activity (Malina 1983), family size and structure (Apraiz 1999), level of educational achievement (Thomas et al. 2001) and altitude (Gonzales et al. 1996). It has been widely observed that age of menarche is lower in more developed European and North American areas than in Africa, Asia and South America. The downward trend toward lower age of menarche found almost universally in studies conducted during the last century has been shown to be reaching a plateau in developed countries, at between 12.5 and 13.5 years (Whincup et al. 2001). Age of menarche continues to decline in developing countries and is expected to do so in line with biosocial development until a similar plateau is achieved (Thomas et al. 2001). Menarcheal age has therefore been used as an indicator of global socio-economic status of a population and the rate of its decline as a proxy measure of developmental growth.

Data regarding age of menarche and trends over time for contemporary rural West African populations are limited. Two such studies have been published in the last decade, one from Senegal and one from Mali, revealing a high median age of menarche in these populations (15.9 years and 14.7 years, respectively) (Pawloski 2002; Garnier et al. 2005). This paper presents a cross-sectional study of the trend in menarcheal age observed in a rural Gambian population over the past three decades. We also present evidence for a rate of decline that is higher than those reported elsewhere, suggesting rapid societal changes.

## Subjects and methods

The three cross-sectional studies were carried out among all female inhabitants aged 9–20 years in three neighbouring villages in rural Gambia, West Africa. Written, informed consent was obtained, with consent gained from a parent or female guardian where the girls were under 16 years. Ethical approvals were obtained from The Joint MRC/Gambian Government Ethics Committee.

The surveys in 2000 and 2008 were conducted by one western female researcher and one Gambian female field worker. In 1989, the survey was conducted by a male western researcher with a female Gambian fieldworker. Girls or their legal guardian were privately asked the question ‘have you seen your moons yet?’ and a yes/no answer recorded. Birth dates were verified by health card and clinic records at MRC (Medical Research Council) Keneba; this clinic has provided routine antenatal care for mothers in these three villages since 1978.

The population of the three villages surveyed is rural, Mandika-speaking West African. Socio-economic status is largely Group V – with most of the community being subsistence farmers with small trading of excess goods. In recent years there has been shift towards a remittance economy, as a result of family members working abroad. There is also some upward mobility of families due to employment by the MRC station located in the region. The diet has changed over the study period from mainly traditional cereal-based foods, to largely rice-based diet. Animal protein intake remained low throughout the study period, but increased amounts of oil began to be used in cooking. Health care is deemed relatively good for such a community, due largely to the presence of an MRC health centre.

Interval regression was used in the main analysis to establish the mean age at menarche for each survey and whether this had changed between the surveys. This analysis also permitted estimation of the standard deviation of the distribution of age at menarche and testing for differences in the standard deviation between surveys. Probit analysis was used to confirm these results and, by fitting the first three orthogonal polynomials in age, to test the assumption that age at menarche followed a normal distribution. The bootstrap was applied to obtain confidence intervals for the mean and standard deviation of the age at menarche from the probit analysis. All analyses were carried out using Stata 9 (Statacorp, College Station, TX, USA).

## Results

**Figure 1.**
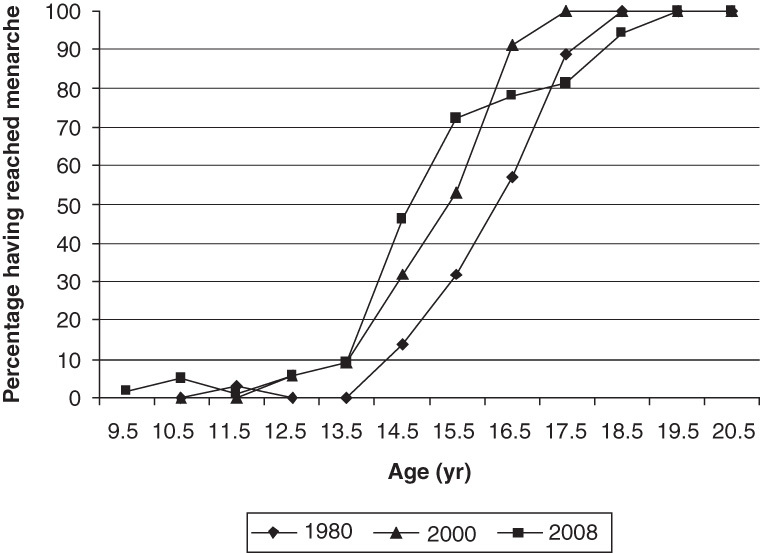
Percentage of girls in a rural Gambian population at three time points who had reached menarche by age.

Of potential eligible subjects for this study numbers of non-participants were 0, in 1989, 9 (3%) in 2000, and 23 (6%) in 2008. This was due to parental or subject non-consent, or because of absence from the village during the study period. The most common cause for absence was residence in the capital city Banjul due to school, work or marriage. Table I and Figure 1 show, for each 1-year age band, the percentage of young women who had reached menarche at the time of study. Table II presents the mean age of menarche estimated from separate interval regression analyses for each survey year. Interval regression of the pooled data for all surveys revealed a significant linear trend towards younger mean age at menarche; it declined by 0.65 (95% CI: 0.37–0.92) years per decade; *p* < 0.00001. This model also yielded an estimate for the standard deviation for the spread of age at menarche of 1.62 (95% CI: 1.45–1.81) years. Since modelling separate means for each survey fitted no better than the linear trend (*p* = 0.16) we have no evidence that this trend is either accelerating or slowing down.

**Table I.** Table showing subject numbers by age, at three time points and percentage of subjects that recorded a ‘yes’ response to having achieved menarche.

Age	Age centre	1989		2000		2008	
		Number of subjects (*n* = 239)	% Yes	Number of subjects (*n* = 346)	% Yes	Number of subjects (*n* = 339)	% Yes
10	10.5	33	0	39	0	30	3
11	11.5	32	3	32	0	40	3
12	12.5	23	0	54	5	39	8
13	13.5	28	0	35	8	27	11
14	14.5	29	14	41	33	30	40
15	15.5	25	32	30	52	26	69
16	16.5	23	57	35	91	44	84
17	17.5	18	89	32	100	27	89
18	18.5	12	100	20	100	14	88
19	19.5	8	100	17	100	18	100
20	20.5	8	100	11	100	14	100

**Table II.** Median age of menarche in a rural Gambian population at three time points.

Time point	Median age at menarche (95% CI)	Standard deviation of age at menarche (95% CI)
1989	16.06 (15.67–16.45)	1.47 (1.15–1.88)
2000	15.03 (14.76–15.30)	1.20 (0.98–1.47)
2008	14.90 (14.52–15.28)	2.00 (1.68–2.37)

Repeating the pooled analysis but with separate estimates of the standard deviation for each survey revealed a significant (*p* = 0.001) difference in the spread of age at menarche between them. This, however, made a negligible difference to the estimate of the linear trend or its precision. Repeating the mean and standard deviation estimation using probit analysis gave almost identical results. Fitting a probit model in which the covariate age was replaced with its first three orthogonal polynomials gave a significant improvement in the model fit (*p* = 0.0009). While this indicates deviation from the underlying assumption that the age at menarche is normally distributed, the effect on estimates of the trend in the mean age at menarche was negligible.

## Discussion

This study revealed the age of menarche of a rural Gambian population to be amongst the highest in the world. The median age of menarche in the year 2000 (15.03 years), places it between the median age of menarche found in other rural West African populations, Mali (14.72 years) and Senegal (15.90 years), in the same year. This contrasts these countries ranking by Human Developmental Index (HDI, a composite measure of life expectancy, adult illiteracy rate and GDP), which ranks Senegal at 153 in the world, The Gambia as 160 and Mali as 168 (Human Development Reports 2008). This reinforces the view that influences on age of menarche are likely to be more multi-factorial than socio-economic status alone.

Age of menarche in this population appears to have declined at amongst the fastest reported rates, at 0.65 years of age per decade. This is comparative to the rates of decline reported from Korea: 0.68 years of age per decade (Cho et al. 2009) and Morocco: 0.55 years of age per decade (Loukid et al. 1996). The observed rate of decline in The Gambia is much faster than that reported for European countries, which has mostly come to a halt, and also faster than reported rates from other African countries; Mozambique: no significant decline in rate (Padez 2003), Cameroon: 0.27 years of age per decade (Pasquet et al. 1999), Ghana: 0.2 years of age per decade (Adanu et al. 2006) and South African Blacks: 0.5 years of age per decade (Jones et al. 2009). This may indicate a population undergoing a period of rapid socio-economic change; a hypothesis borne out by a concurrent improvement in HDI amongst the highest in the world. Between 1975 and 2005 The Gambia's HDI improved from 0.29 to 0.50, an increase only rivalled by India, China, Nepal, Korea, Saudi Arabia, Oman, Indonesia, Morocco and Algeria (Human Development Reports 2008). It is interesting to note that Morocco and Korea, both countries with rates of decline in menarcheal age similar to The Gambia, also have had a similarly rapid improvement in HDI.

This study had a relatively high participation rate of greater than 93%. However, there may have been a small selection bias in the data because of the possibility that girls who had reached menarche may have been deemed more mature and hence more likely to have migrated outside of the study area for marriage or work. Discussing this possibility with residents of the area, however, gave little anecdotal evidence that this was the case.

As with any observational study, the causal factors for the decline in age of menarche cannot be identified. However, the coincidental increase in socio-economic indices occurring in The Gambia during the period of study mirrors findings from other countries, suggesting that rate of decline of median menarcheal age, if not the age itself, may have use as a marker of rate of socio-economic development of a country.
